# High-Fat Diet Affects Heavy Metal Accumulation and Toxicity to Mice Liver and Kidney Probably via Gut Microbiota

**DOI:** 10.3389/fmicb.2020.01604

**Published:** 2020-07-28

**Authors:** Ting Liu, Xue Liang, Chao Lei, Qinhong Huang, Weiqi Song, Rong Fang, Chen Li, Xiaomei Li, Hui Mo, Ning Sun, Haoran Lv, Zhihua Liu

**Affiliations:** ^1^Guangzhou Key Laboratory of Enhanced Recovery after Abdominal Surgery, The Fifth Affiliated Hospital of Guangzhou Medical University, Guangzhou, China; ^2^The First Affiliated Hospital of Guangzhou Medical University, Guangzhou, China; ^3^Department of Public Health, Guangzhou Medical University, Guangzhou, China; ^4^The Public Laboratory, South China Botanical Garden, Chinese Academy of Sciences, Guangzhou, China; ^5^Research Institute of Chinese Medicine Sciences, Guangdong Pharmaceutical University, Guangzhou, China

**Keywords:** high-fat diet, heavy metal, gut microbiota, arsenic, cadmium, lead

## Abstract

Previous studies proved that heavy metals could increase the risk of disease by acting on the gut microbiota. Meanwhile, gut microbiota played important roles in detoxifying heavy metals. However, the response of gut microbiota to heavy metals and which microbes dominated this detoxification processes are still unclear. This study investigated the difference of high-fat-diet (HFD) and normal-diet (ND) gut microbiota and their response to and detoxification effects on arsenic (As), cadmium (Cd), and lead (Pb) exposure. Results showed that gut microbiota of ND and HFD was significantly different and responded to As, Pb, and Cd exposure differently, too. When exposed to 100 ppm As, Cd, or Pb, HFD-fed mice accumulated more heavy metals in the liver and kidney along with more severe functional damage than ND-fed mice, indicated by a more dramatic increase of alanine aminotransferase (ALT) and aspartate aminotransferase (AST) activities and urinary total protein (TPU), urinary uric acid (UUA), and urinary creatinine (Ucrea) content. Among ND gut microbiota, relative abundance of *Bacteroides*, *Lactobacillus*, *Butyricimonas*, and *Dorea* was significantly increased by arsenic (As) exposure; relative abundance of *Faecoccus* and *Lactobacillus* was significantly increased by Cd exposure; relative abundance of *Desulfovibrio*, *Plasmodium*, and *Roseburia* were significantly increased by Pb exposure. However, among HFD gut microbiota, those microbes were not significantly changed. Bivariate association analysis found weak positive correlations between content of fecal excreted heavy metals and richness of total fecal microbiota as well as abundance of some of the heavy metal-enriched microbes. Our study concluded that HFD increased disease risk of heavy metal exposure probably via its gut microbiota which excreted less heavy metal through feces.

## Introduction

Environmental toxin exposure is a global health problem in the 21st century. Heavy metals are one of the most harmful environmental toxins, which are widely found in polluted air, water, and soil. Heavy metals enter and accumulate gradually in the human body through diet uptake. Numerous studies have shown that heavy metal pollution is widely spread in animal and plant products, aquatic products, and various processed foods all over the world (Dadar et al., 2016; Liu et al., 2016; Wijayawardena et al., 2016). Heavy metals that enter the human body through chronic exposure are very difficult to metabolize or decompose, so they accumulate in all tissues and organs over the years and exert chronic damage to the body when they reach a certain threshold (Raehsler et al., 2018). Epidemiological investigations have shown that heavy metals can be detected in the blood, urine, hair, and nails of healthy and diseased people and that the content is correlated with severity of respiration diseases (Wu et al., 2018), cardiovascular diseases (Lamas et al., 2016), neurodegeneration (Bjorklund et al., 2018a; Ghazala et al., 2018; Iqbal et al., 2018) diseases, autism spectrum disorder (Bjorklund et al., 2018b), and obesity (Park et al., 2017; Shao et al., 2017; Wang et al., 2018). Great efforts have been made to reduce heavy metal pollution, but these efforts often have very limited effects (Bisanz et al., 2014). So it is an urgent requirement to explore new methods to reduce their health risks.

Gut microbiota are the most important microecosystem in the human body, which chemically modifies dietary compounds, industrial compounds, pollutants, and drugs, thereby affecting their disease risk, bioavailability, toxicity, and efficacy. However, how gut microbiota metabolize these substances and their impact on human health are still unclear (Koppel et al., 2017). The mechanism of how gut microbiota detoxify heavy metals was rarely reported. More Cd or Pb was detected in the blood and organs such as liver, kidney, and spleen in germ-free mice compared to specific pathogen-free mice after drinking water containing inorganic Cd or Pb for 6 weeks (Breton et al., 2013a, b), indicating that the gut microbiota of SPF mice prevented heavy metals from entering the bloodstream through the gastrointestinal tract and accumulating in target organs. Feces from rat could transfer methylmercury into inorganic mercury, which was less toxic and easier to excrete (Rowland et al., 1978). When rat gut microbiota was eliminated by antibiotics, methylmercury accumulated more, and neurotoxic symptoms became more serious (Rowland et al., 1980). Studies have reported the presence of genes in intestinal microbes related to heavy metal metabolism. Mercury-resistant gene operons were discovered in gram-negative bacteria in primate fecal microbiota (Liebert et al., 1997). Deep excavation of human microbiome data revealed that there were symbiotic microbes in the gastrointestinal tract containing all known genes encoding arsenic-sensing and regulating proteins (Isokpehi et al., 2014). Together, these data implied that the gut microbiota could detoxify heavy metals through absorption, metabolism, sequestration, and excretion. However, response of gut microbiota to heavy metals and which microbes dominated this detoxification processes were still unclear.

Gut microbiota are modified by heredity and various environmental factors, among which diet is the major determinant (Thomas et al., 2017). High-fat diet (HFD) is a common problem worldwide, and the gut dysbiosis caused by HFD is closely related to the incidence of various diseases including obesity, diabetes, cardiovascular disease, and tumor (Cordain et al., 2005). Epidemiological studies have shown that obese people, the majority of which have HFD, accumulate more heavy metals in their bodies than do healthy people (Park et al., 2017; Shao et al., 2017; Wang et al., 2018). We propose that gut microbiota of HFD might have weaker ability to eliminate or detoxify heavy metals than gut microbiota of normal diet (ND). This study investigated the difference of gut microbiota between HFD- and ND-fed mice and their detoxification effects on As, Cd, and Pb exposure. Efforts were made to find characteristics of gut microbiota that have a positive correlation with heavy metals that are excreted more in feces, accumulated less, and damaged the liver and kidney more mildly as well as specific microbes that tolerated heavy metals and may have roles in detoxifying As, Cd, and Pb.

## Results

### Diet Effects on Liver Function Damage Upon Heavy Metal Exposure

**FIGURE 1:**
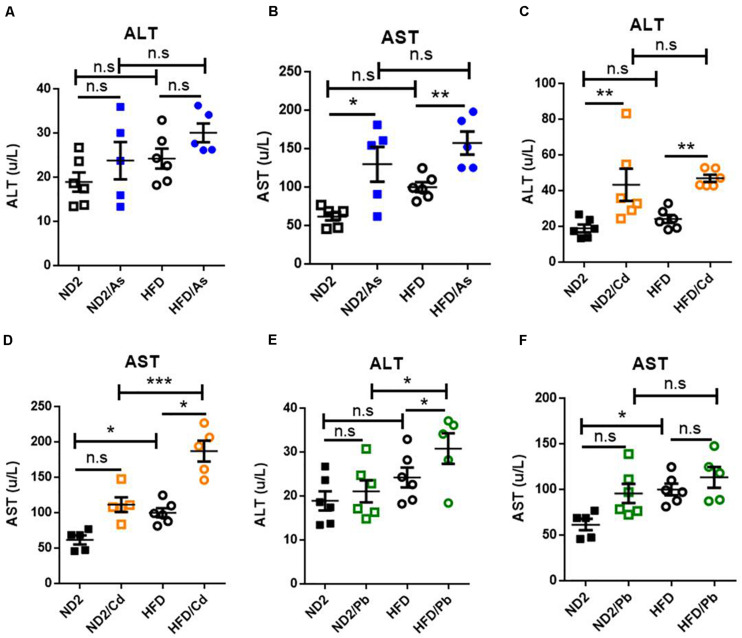
Diet effects on liver function damage upon heavy metal exposure. **(A–F)** ND- and HFD-fed mice were exposed to 100 ppm As, Cd, or Pd for 10 weeks (*n* = 6 for each group). Plasma was collected and analyzed for AST and ALT activities on the day of killing. Data were expressed as mean ± SEM. Statistics according to two-way ANOVAs and Bonferroni *post hoc* tests. n.s. not significant; *P < 0.05; **P < 0.01; ***P < 0.001. ALT, alanine aminotransferase; AST, aspartate aminotransferase.

To determine the impact of dietary patterns on mice’s response to heavy metal exposure, we first analyzed activity of blood alanine aminotransferase (ALT) and aspartate aminotransferase (AST), the two primary indicators of liver function in plasma samples. Higher activities of ALT or AST indicate more severe liver damage. Arsenic (As), Cd, or Pb was used to treat both ND- and HFD-fed mice. Results showed that consuming HFD versus ND led to higher activity of AST and ALT and that heavy metals further increased the activity of AST and ALT. So HFD-fed mice exposed to heavy metals had the highest AST and ALT activities among all the groups (Figures 1A–F). In ND-fed mice, only AST upon As exposure and ALT upon Cd exposure increased significantly (*P* < 0.05) (Figures 1B,C), while in HFD-fed mice, AST upon As exposure (Figure 1B), ALT and AST upon Cd exposure (Figures 1C,D), and ALT upon Pb exposure (Figure 1E) increased significantly. Together, these results indicated a possibility that compared to ND, HFD increased liver damage caused by As, Cd, and Pb exposure.

### Diet Effects on Kidney Functional Damage Upon Heavy Metal Exposure

**FIGURE 2:**
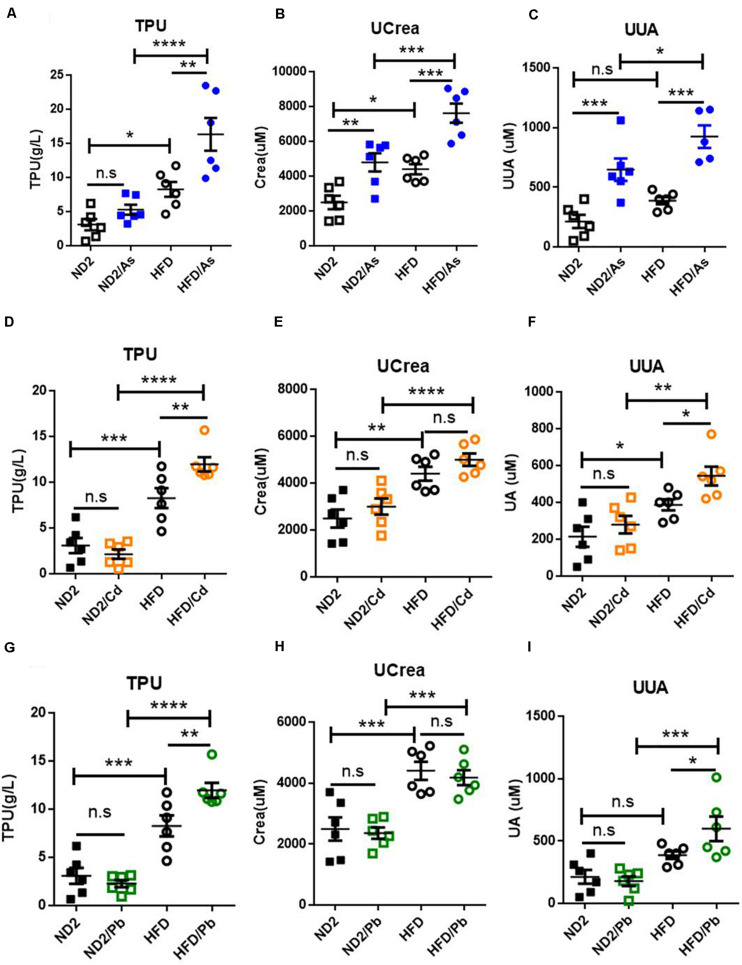
Diet effects on kidney function damage upon heavy metal exposure. **(A–I)** ND- and HFD-fed mice were exposed to 100 ppm As, Cd, or Pd for 10 weeks (*n* = 6 for each group). Urine was collected and analyzed for total protein, uric acid, and creatinine contents on the day of sampling. Data were expressed as mean ± SEM. Statistics according to two-way ANOVAs and Bonferroni *post hoc* tests. n.s. not significant; *P < 0.05; **P < 0.01; ***P < 0.001; ***P < 0.0001. TPU, urinary total protein; UUA, urinary uric acid; Ucrea, urinary creatinine.

To further determine whether HFD would increase kidney damage of heavy metals, we analyzed urine total protein (TPU), urinary creatinine (UCrea), and urinary uric acid (UUA) contents, the three main indicators of kidney function, with higher contents indicating a heavier functional damage. Results showed that consuming HFD versus ND led to higher content of TPU, UCrea, and UUA and that heavy metals further increased content of TPU, UCrea, and UUA with mostly more increase in HFD- versus ND-fed mice (Figures 2A–I). Meanwhile, in ND-fed mice, only UCrea and UUA upon As exposure increased significantly (Figures 2B,C), while in HFD-fed mice, all three indicators increased significantly upon As exposure (Figures 2A–C), TPU and UUA increased significantly upon Cd exposure (Figures 2D,F), and TPU and UUA increased significantly upon Pb exposure (Figures 2G,I). Those results implied a trend that HFD increased kidney damage caused by As, Cd, and Pb exposure.

### Diet Effects on Heavy Metal Accumulation in Liver and Kidney and Elimination in Feces

**FIGURE 3:**
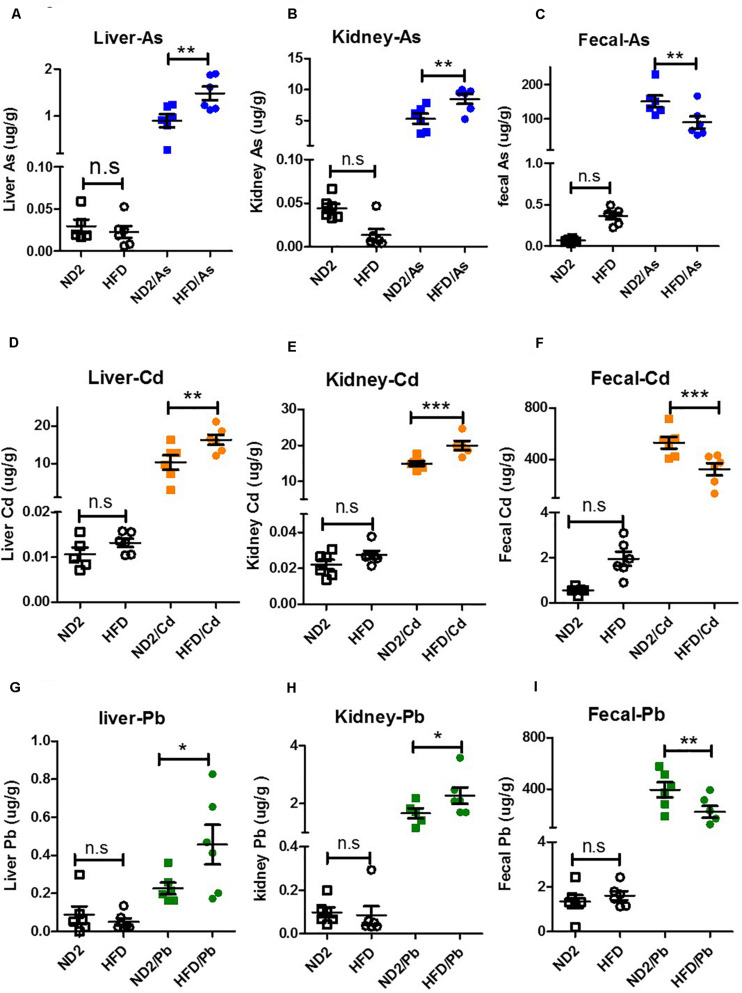
Diet effects on heavy metal accumulation in the liver and kidney and elimination in feces. ND- and HFD-fed mice were exposed to 100 ppm As, Cd, or Pd for 10 weeks (*n* = 6 for each group). Feces, liver, and kidney were collected for As, Cd, or Pb content analysis. **(A,B)** Bioaccumulation of As in the liver/kidney of ND- and HFD-fed mice upon As exposure. **(C)** As content in feces of ND- and HFD-fed mice upon As exposure. **(D,E)** Bioaccumulation of Cd in the liver/kidney of ND- and HFD-fed mice upon Cd exposure. **(F)** Cd content in feces of ND- and HFD-fed mice upon Cd exposure. **(G,H)** Bioaccumulation of Pb in the liver/kidney of ND- and HFD-fed mice upon Pb exposure. **(I)** Pb content in feces of ND- and HFD-fed mice upon Pb exposure. Data were expressed as mean ± SEM. Statistics according to two-way ANOVAs and Bonferroni *post hoc* tests. n.s. not significant; *P < 0.05; **P < 0.01; ***P < 0.001.

To examine the impact of diet on the accumulation of heavy metals in mice, the As, Cd, and Pb content in livers, kidneys, and fecal samples was investigated in ND- and HFD-fed mice exposed to As, Cd, or Pb (Figures 3A–I). As expected, upon As, Cd, or Pb exposure, these heavy metals accumulated significantly more in livers and kidneys in HFD-fed mice than in ND-fed mice when the background accumulation in ND- and HFD-fed mice with no heavy metal treatment had no significant difference or was even lower (Figures 3A,B,D,E,G,H). Interestingly, upon As, Cd, or Pb exposure, these heavy metals were excreted significantly less through feces in HFD-fed mice compared to ND-fed mice while their background content in feces of mice with no heavy metal treatment had no significant difference (Figures 3C,F,G). These data implied that higher accumulation of heavy metals in organs of HFD-fed mice might result from less fecal excretion.

### Diet Effects on Total Gut Microbiota Disturbance Upon As, Cd, or Pb Exposure

Since different dietary patterns shape unique gut microbiota profiles, to explore whether gut microbiota played a role in explaining the different harmful effects of heavy metals on ND- and HFD-fed mice, we analyzed the difference of microbiota in fecal samples of ND- and HFD-fed mice. A phylogenetic tree was made to summarize the observed alterations in relative abundance of microbial taxa (Supplementary Figure S1). It showed that there was a significant difference between gut microbiota of ND- and HFD-fed mice, which may respond differently to heavy metals.

**FIGURE 4:**
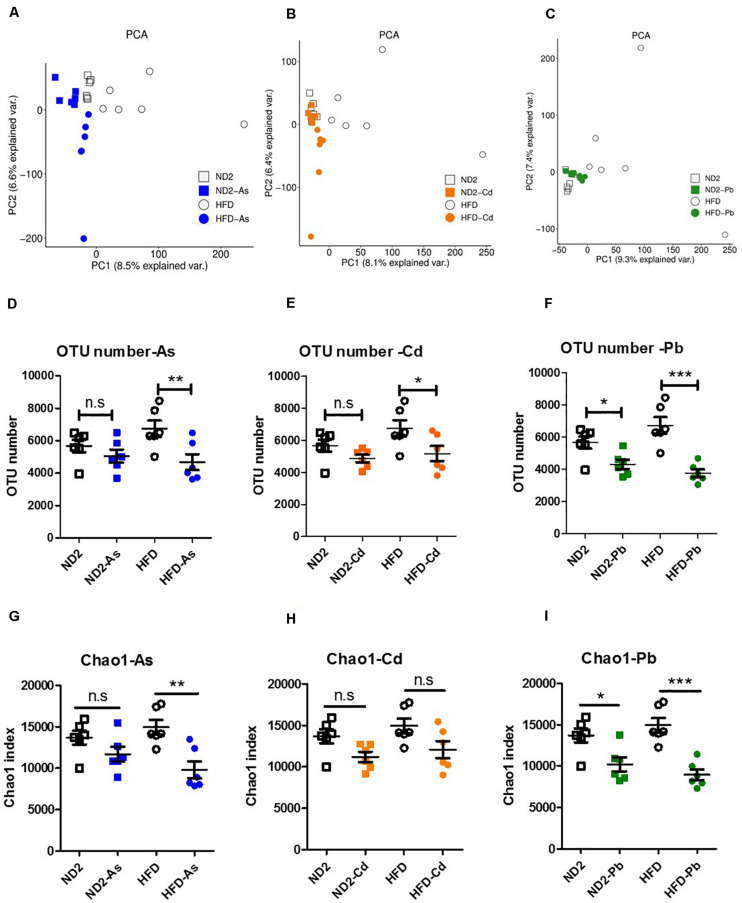
Diet effects on gut microbiota disturbance upon As, Cd, or Pb exposure. ND- and HFD-fed mice were exposed to 100 ppm As, Cd, or Pd for 10 weeks (*n* = 6 for each group). Cecum feces was collected for gut microbiota analysis by 16S rRNA gene sequencing. **(A–C)** Principal coordinate analysis (PCA) based on an unweighted UniFrac analysis of the intestinal microbial composition where samples of ND-/HFD-fed mice upon As, Cd, or Pb exposure are highlighted with different colors. The position and distance of data points indicate the degree of similarity in terms of both presence and relative abundance of bacterial taxonomies. **(D–F)** Number of observed OTUs. Data (mean + SEM) represent 16S rRNA gene 454-pyrosequencing analysis of intestinal microbiota of mice. **(G–I)** Chao1 index of microbial diversity. Statistics according to two-way ANOVAs and Bonferroni *post hoc* tests. n.s. not significant; *P < 0.05; **P < 0.01; ***P < 0.001.

As expected, the gut microbiota of ND- and HFD-fed mice respond differently to heavy metals. Principal coordinate component analysis (PCA) based on an unweighted UniFrac analysis showed that the gut microbiota of both ND- and HFD-fed mice changed upon As, Cd, or Pb exposure. However, the HFD microbiota changed more than the ND microbiota (Figures 4A–C). Arsenic (As), Cd, or Pb exposure reduced microbiota abundance based on operational taxonomic unit (OTU) number (Figures 4D–F) and microbial diversity based on the Chao1 index (Figures 4G–I) in both ND- and HFD-fed mice; however, there was more reduction in microbiota abundance and diversity in HFD- than in ND-fed mice. Generally, these data indicated that gut microbiota of HFD-fed mice suffered more disturbance upon As, Cd, or Pb exposure.

### Diet Effects on Specific Gut Microbiota Genus Upon As, Cd, or Pb Exposure

**FIGURE 5:**
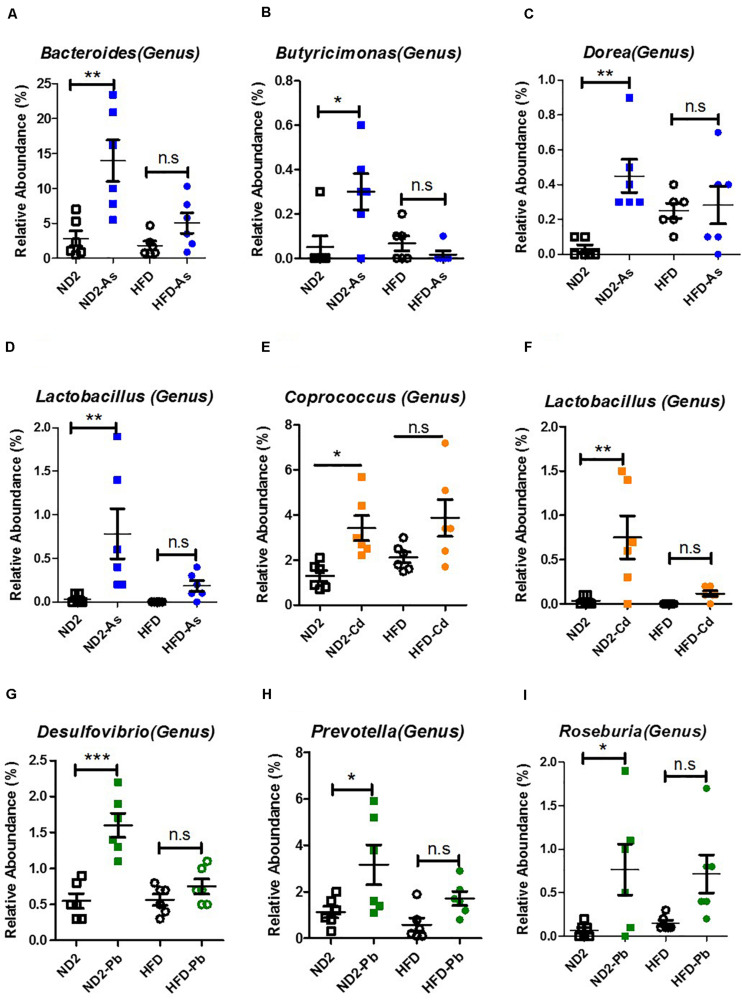
Microbial genus enriched significantly by heavy metals primarily in ND-fed mice. ND- and HFD-fed mice were exposed to 100 ppm As, Cd, or Pd for 10 weeks (*n* = 6 for each group). Cecum feces was collected for gut microbiota analysis by 16S rRNA gene sequencing. **(A)** *Bacteroides* in the Bacteroidetes phylum, Bacteroidia class, Bacteroidales order, Bacteroidaceae family. **(B)** *Butyricimonas* in the Bacteroidetes phylum, Bacteroidia class, Bacteroidales order, Odoribacteracea family. **(C)** *Dorea*, **(E)** *Coprococcus*, and **(I)** *Roseburia* in Firmicutes phylum, Clostridia class, Clostridiales order, Lachnospiraceae family. **(D,F)** *Lactobacillus salivarius* in the Firmicutes phylum, Bacilli class, Lactobacillales order, Lactobacillaceae family. **(G)** *Desulfovibrio* in Proteobacteria phylum, Deltaproteobacteria class, Desulfovibrionales order, Desulfovibrionaceae family. **(H)** *Prevotella* in Bacteroidetes phylum, Bacteroidia class, Bacteroidales order, Prevotellaceae family. Data are expressed as mean ± SEM. Statistics according to two-way ANOVAs and Bonferroni *post hoc* tests. n.s. not significant; *P < 0.05; **P < 0.01; ***P < 0.001.

To further find specific microbiota that were changed by heavy metals and which one(s) may confer detoxification effects, we analyzed the abundance change of gut microbiota at all taxon levels by arsenic (As), Cd, or Pb treatment in both ND- and HFD-fed mice (Supplementary Figures S2–S7). Specific gut microbiota genus that was changed significantly by As, Cd, or Pb treatment was summarized in Supplementary Table S2. Interestingly, among all the specific microbiota genera that were changed by heavy metals, a few increased significantly in the microbiota of ND- but not HFD-fed mice. Upon arsenic (As) exposure, *Bacteroides*, *Butyricimonas*, *Dorea*, and *Lactobacillus* increased significantly in the microbiota of ND- but not HFD-fed mice (Figures 5A–D). Upon Cd exposure, *Coprococcus* and *Lactobacillus* increased significantly in microbiota of ND- but not HFD-fed mice (Figures 5E,F). Upon Pb exposure, *Desulfovibrio*, *Prevotella*, and *Roseburia* increased significantly in microbiota of ND- but not HFD-fed mice (Figures 5G–I). These gut microbiota genera that were enriched by heavy metals in ND-fed mice that suffered less toxicity and accumulation might be candidate microbes with detoxification ability.

### Correlation Between Fecal As, Cd, and Pb Content and Abundance of Total Gut Microbiota and Specific Microbiota Genus

**FIGURE 6:**
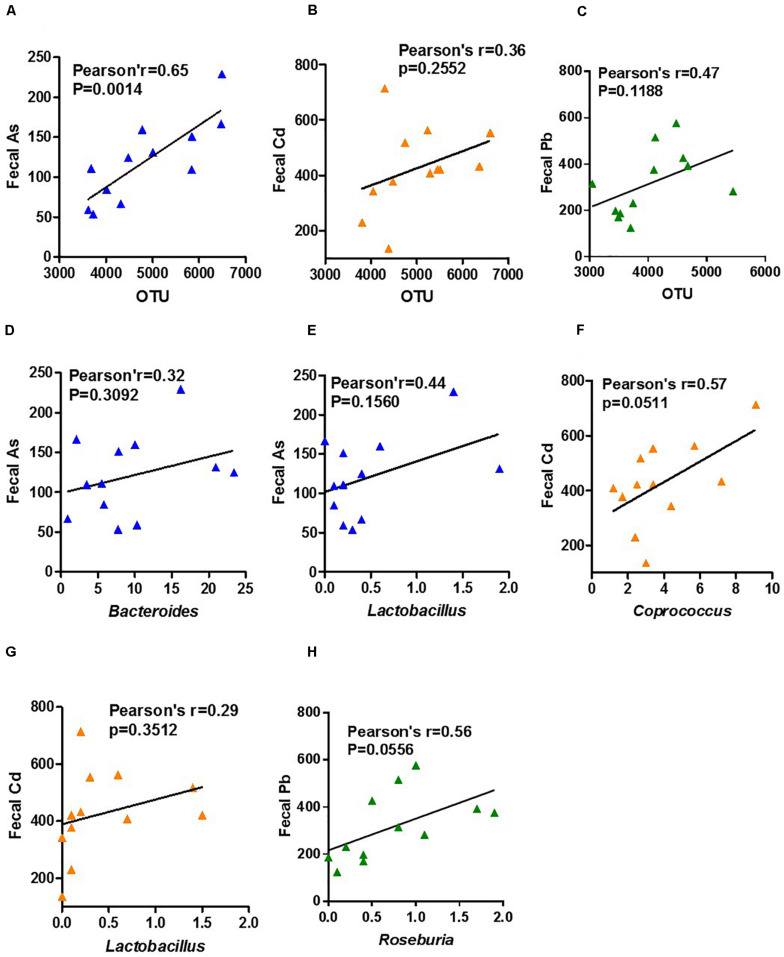
Correlation between fecal As, Cd, and Pb content and gut microbiota abundance of total and specific microbiota genera. Data of ND- and HFD-fed mice upon As, Cd, or Pb exposure were used for Pearson’s correlation analysis. Bivariate scatterplots between **(A)** fecal As and total microbiota abundance of As-exposed mice, **(B)** fecal Cd and total microbiota abundance of Cd-exposed mice, **(C)** fecal Pb and total microbiota abundance of Pb exposed mice, fecal As and *Bacteroides* **(D)** and *Lactobacillus* **(E)** abundance of As-exposed mice, fecal Cd and *Coprococcus* **(F)** and *Lactobacillus* **(G)** abundance of Cd-exposed mice, and **(H)** fecal Pb and *Roseburia* abundance of Pb-exposed mice.

To further evaluate the possible detoxification role of the above gut microbiota genera that were enriched by heavy metals in ND-fed mice, we analyzed the correlation between the abundance of these specific microbiota genera as well as the abundance of total gut microbiota and content of fecal heavy metals, based on an assumption that gut microbiota especially those specifically enriched microbiota genera might help to excrete heavy metals with feces, which results in less heavy metal accumulation and organ damage. Results showed that the total microbe abundance and fecal As, Cd, and Pb concentrations were positively correlated, among which microbe abundance of As-exposed mice and their fecal As were significantly positively correlated (Figures 6A–C). Weak positive correlations were also found between arsenic (As)-enriched *Bacteroides* and *Lactobacillus* and fecal As content (Figures 6D,E), Cd-enriched *Coprococcus* and *Lactobacillus* and fecal Cd content (Figures 6F,G), and Pb-enriched *Roseburia* and fecal Pb content (Figure 6H). These results implied a causal link between those microbes and heavy metal detoxification.

## Discussion

It is widely accepted that diet is the most important determinant of gut microbiota (Lynch and Hsiao, 2019). Studies have confirmed that gut microbiota played roles in heavy metal-caused diseases (Ba et al., 2017; Kim et al., 2017). Tinkov et al. (2018) proposed a causal link between Cd exposure, gut microbiota, and diseases: Cd exposure acted on the gut microbiota and intestines, resulting in increased intestinal barrier permeability, local intestinal inflammation, changes in intestinal microbiota, and their metabolic functions, which led to bacterial translocation, increased lipopolysaccharides and endotoxins, and eventually the risk of infectious diseases. Moreover, metabolic disorders and systemic inflammation would act on target organs such as adipose tissue, liver, kidney, cardiovascular system, and brain, which would increase the risk of corresponding diseases (Tinkov et al., 2018). Here, we report that HFD affects heavy metal toxicity and accumulation in mice liver and kidney and report its relationship to gut microbiota. Results showed that when exposed to heavy metals, there was more heavy metal accumulation in the liver and kidney of HFD- versus ND-fed mice and organ function damage increased more in HFD-fed compared to ND-fed mice. It is probably because of their distinct gut microbiota: HFD microbiota suffered more disturbances when exposed to heavy metals. Some microbes that could respond to heavy metals and enrich them in ND-fed mice did not respond or cannot enrich as much in HFD-fed mice. The abundance of overall microbiota and some of these specific microbes had a positive correlation with the content of fecal heavy metals, suggesting that the gut microbiota especially the specific microbes might play a role in heavy metal detoxification probably via absorption and fecal excretion.

A few literatures reported changes of gut microbiota caused by heavy metal exposure (reviewed by Gillois et al., 2018). There were no consistent results about microbiota changes in each study at the taxa level of species, genus, family, order, or even phylum, which may result from differences in dosage of the heavy metals, exposure time, physiological feature of experimental animals, and most importantly, the food composition of the diet (Tinkov et al., 2018). Anyhow, different intestinal microbes responded differently to heavy metal stresses, and their maximum tolerated concentrations and sorption properties were different (Riley and Mee, 1982; Sizentsov et al., 2019). Our *in vivo* experiments in mice showed that under 100 ppm As, Cd, or Pb, most gut microbes were tolerant without abundance change while a few were sensitive indicated by a decrease in abundance (Supplementary Figures S2–S7 and Supplementary Table S2), implying that some intestinal microbes might not respond to stresses from specific heavy metals. Moreover, we found that the same heavy metal caused mostly the same change of the same microbes in both ND and HFD. For example, under arsenic exposure, *Dorea*, *Lactobacillus*, and *Bacteroides* all increased while *Allobaculum*, *Akkermansia*, and *Bilophila* all decreased in both ND- and HFD-fed mice; however, a few microbes such as *Butyricimonas*, *Adlercreutzia*, and *Prevotella* showed opposite changes in ND- and HFD-fed mice. This may be due to the mutual promotion or inhibition of individual microbes in a complex microbiota ecosystem, which affects their response to stress.

It is worth noting that in five out of six heavy metal treatment groups, *Lactobacillus* was significantly increased, and *Akkermansia* was significantly decreased, regardless of feeding diet (Supplementary Table S2). *Lactobacillus* abundance was positively correlated with fecal discharge of both As and Cd. With an estimated >200 species presently, *Lactobacillus* is well known for its ability to modulate the microbiota in the host gastrointestinal tract, conferring beneficial effects in health (Holzapfel and Wood, 2014; Yeo et al., 2018). Our study proposed that *Lactobacillus* might also have function in heavy metal detoxification. The decrease in *Akkermansia* had been shown in various disease conditions such as obesity and inflammatory bowel disease (Naito et al., 2018). Our study indicated that its decrease might also be related to the risk of disease caused by heavy metal exposure.

Several other gut microbes including *Bacteroides*, *Coprococcus*, and *Roseburia* were enriched by heavy metals and were also positively correlated with heavy metal content in feces. Genomic and subsequent proteomic analyses for *Bacteroides thetaiotaomicron* and *Bacteroides fragilis* have found multiple pump systems to get rid of toxic substances (Wexler, 2007). *Roseburia* spp. were commensal bacteria producing short-chain fatty acids and could serve as markers of human health (Tamanai-Shacoori et al., 2017). Although their correlations were weak, they provided candidates for future screening for heavy metal detoxification. Certainly, the roles and mechanisms of these intestinal microbes in detoxifying heavy metals and reducing their toxicity to the host still needed to be verified by *in vivo* single-strain replenishment experiments. Since with sequence of 16S rRNA V3/V4, we could not annotate gut microbes accurately to species level, it was expected that full-length sequence could screen out intestinal microbes at the species level that respond to heavy metals in future studies; then proper strains could be used for experimental verification, and the detoxification mechanism has to go after these efforts.

Health risks of heavy metal exposure could be reduced by interventions targeting gut microbiota. Cd-exposed rats when fed with probiotics *Lactobacillus plantarum* and *Bacillus coagulans* showed reduced Cd accumulation in the liver and kidney, as well as reduced serum alanine transaminase and glutamic transaminase activities and creatinine and urea concentrations, suggesting a reduction in Cd toxicity (Jafarpour et al., 2017). In rats exposed to Cd, the abundance of lactic acid bacteria in fecal microbiota was positively correlated with fecal Cd content and negatively correlated with the serum Cd content, indicating that the probiotics might absorb Cd and exclude it (Djurasevic et al., 2017). A randomized open-label pilot study in heavy metal-contaminated areas showed that supplementation of *Lactobacillus rhamnosus* resulted in significantly lower levels of mercury (Hg) and arsenic (As) in the blood of pregnant women than in the control group (Bisanz et al., 2014), suggesting that interventions targeting gut microbiota in specific populations may reduce the health risks of heavy metals. Our study showed that different diets affected accumulation of heavy metal and damage of liver and kidney after heavy metal exposure. It is reasonable to test the possibilities that health risk of heavy metal exposure might be reduced through dietary interventions targeting gut microbiota.

## Materials and Methods

### Mice

Animal experiments were approved and performed in accordance with the guidelines of the Laboratory Animal Center of Guangzhou Medical University (animal protocol number: 2019-634). Eight-week-old male C57BL/6 mice were purchased from Guangdong Medical Laboratory Animal Center (GDMLAC) and kept under controlled temperature and light conditions (25°C, 12-h light–dark cycle), with free access to food and water. Mice were randomly divided into eight groups containing six animals each and housed in groups of three animals per cage. Four groups of mice were fed with ND (13.5% of energy from fat; D12450; GDMLAC, China) and the other four groups with HFD (45% of energy from fat; D12451; GDMLAC, China). The formula of the diet was shown in Supplementary Table S1. Each group of ND-/HFD-fed mice was given 0/100 ppm As (NaAsO_2_, Merck), Cd (CdCl_2_.5H_2_O, Macklin), or Pd (PbCl_2_, Macklin) in drinking water for 10 weeks.

At week 10, urine and fecal samples were taken. Fecal samples were dried in an oven at 65°C for 24–30 h until the weight became constant. The weight of dried fecal sample was recorded. Animals were fasted for 12 h before killing. Mice were deeply anesthetized with 1% pentobarbital sodium (50 mg/kg BW), and whole blood was withdrawn through the ventral aorta in tubes containing anticoagulant KEDTA. The kidney and liver were removed and weighed. Feces in the cecum was squeezed out. Organ and cecum content samples were immersed in liquid nitrogen and stored at −80°C for further analysis.

### Liver Function Analysis

Whole blood was withdrawn through the ventral aorta in tubes containing anticoagulant K2EDTA. Blood was centrifuged at 500 *g* for 5 min, and supernatants (plasma) were collected. Two biomarkers of liver function, activity of ALT and AST, were determined on the day of sampling by commercial ELISA kits: ALT (Cat #05850797190, Roche Diagnostics, United States) and AST (Cat #05850819190, Roche Diagnostics, United States), according to the manufacturer’s instructions.

### Kidney Function Analysis

Three biomarkers of kidney function, TPU, UUA, and Ucrea, were determined on the day of sampling by commercial ELISA kits: total protein (Cat #051718190, Roche Diagnostics, United States), uric acid (Cat #05171857190, Roche Diagnostics, United States), and creatinine (Cat #06407137190, Roche Diagnostics, United States) according to the manufacturer’s instructions.

### Gut Microbiota Analysis

Cecal microbiota DNA was extracted using a Stool DNA Kit (Guangzhou IGE Biotechnology, China) and applied to amplification of V3–V4 regions of 16S rDNA. Cecal microbiota composition was assessed using Illumina 2500 sequencing of 16S rDNA amplicon and QIIME-based microbiota analysis. High-quality reads for bioinformatics analysis were selected, and all of the effective reads from all samples were clustered into OTUs based on 99% sequence similarity according to QIIMEU clust. OTUs were annotated through the RDP Classifier (Version 2.2), with a confidence cutoff of 0.8 according to the Green Gene database, and the composition and abundance information of each sample at different classification levels were statistically summarized. Based on the OTU information from each sample, PCA was applied via R to examine the similarity between different samples; α-diversity analyses were performed via the R package phyloseq v.1.19.1 and vegan 2.4.2 packets to calculate the diversity index.

### Heavy Metal Analysis

Dried fecal samples and frozen liver and kidney were digested with 6 ml 65% HNO_3_ (Merck Darmstadt, Germany) at 25°C overnight and further digested by a microwave sample preparation system (Multiwave 3000, Anton Paar, Austria). After digestion, the solutions were diluted with ultrapure water to a final volume of 50 ml. As, Pb, and Cd were measured by inductively coupled plasma mass spectrometry (7700×, Agilent, Japan) according to Sanchez Lopez et al. (2003).

### Statistical Analysis

Required sample sizes were calculated to obtain a power of 0.8, based on the results of similar, previous studies and preliminary data from our own laboratory. Statistical analysis was performed using GraphPad Prism Version 7.0. Unless otherwise indicated, data were analyzed by two-way (heavy metals × diet) repeated-measures ANOVA, followed by Bonferroni *post hoc* tests. All data were presented as mean ± SD. *P*-values < 0.05 were considered significant (^∗^*P* < 0.05, ^∗∗^*P* < 0.01, ^∗∗∗^*P* < 0.001). Graphs were generated using the software GraphPad Prism 7.0. All significant *post hoc* test results are depicted in the figures. Bivariate associations between fecal heavy metal content and the whole and genus-level gut microbiota abundance were assessed using Pearson’s correlation.

## Conclusion

Gut microbiota could detoxify environment toxins through absorption and/or metabolic conversion (Koppel et al., 2017). Intervention targeting gut microbiota to reduce health risks of heavy metals was expected to be safe and cost-effective (Bisanz et al., 2014). To explore the responses of the gut microbiota upon heavy metal stress, exploiting the strains with the potential to detoxify heavy metals and clarifying the detoxification mechanism were the premise of the method. Our study showed that when exposed to 100 ppm As, Cd, or Pb, HFD-fed mice accumulated more heavy metals in the liver and kidney along with more severe function damage than did ND-fed mice, which might result from lower abundance and diversity of HFD microbiota, especially specific gut microbes that might be involved in this detoxification process. Therefore, the health risk of heavy metal exposure might be reduced through dietary interventions targeting gut microbiota. Arsenic-enriched *Bacteroides* and *Lactobacillus*, cadmium-enriched *Coprococcus* and *Lactobacillus*, and lead-enriched *Roseburia* were positively correlated with As, Cd, or Pb content in stools, respectively, and accumulated less in organs, suggesting that these might be the microbes with the potential to detoxify heavy metals. However, the role of these intestinal microbes in detoxifying heavy metals still needs to be verified by future *in vivo* single-strain replenishment experiments.

## Data Availability Statement

The datasets generated for this study can be found in the Sequence Read Archive (SRA), PRJNA596575.

## Ethics Statement

The animal study was reviewed and approved by the Committee Review of Animal Experiments in Guangzhou Medical University (Document no. 2019-634).

## Author Contributions

TL, XL, ZL, and HL conceived and designed the experiments. TL, CLe, QH, CLi, XL, HM, and RF carried out the experiments. TL and WS participated in analyzing the data. TL and NS drafted the manuscript. All authors read and approved the final version of the manuscript.

## Conflict of Interest

The authors declare that the research was conducted in the absence of any commercial or financial relationships that could be construed as a potential conflict of interest.

## Supplementary Material

FIGURE S1 HFD changes relative abundance of most intestinal microbial taxa. Cecum fecal of ND- and HFD-fed mice (*n* = 6 for each group) was collected for gut microbiota analysis by 16S rRNA gene sequencing. Phylogenetic tree created manually showing specific differences in intestinal microbial community at different taxonomic levels between ND and HFD-fed mice. Nodes represent taxa, and the size of each node represents its relative abundance. The color red indicates an increase, blue indicates a decrease, and black indicates no change of relative abundance in HFD compared with ND fed mice. The fully colored nodes indicate the statistically significant difference (*P* < 0.05) and the hollow nodes indicate the statistically non-significant difference by unpaired two-tailed student’s *t*-test.

FIGURE S2 As exposure changes relative abundance of specific intestinal microbial taxa in ND-fed mice. ND-fed mice were exposed to 100 ppm As for 10 weeks (*n* = 6 for each group). Cecum fecal was collected for gut microbiota analysis by 16S rRNA gene sequencing. Phylogenetic tree created manually showing specific differences in intestinal microbial community at different taxonomic levels between As exposure and control mice. Nodes represent taxa, and the size of each node represents its relative abundance. The color red indicates an increase, blue indicates a decrease, and black indicates no change of relative abundance in As exposure compared with control mice. The full color of the nodes indicates the statistically significant difference and the hollow nodes indicate the statistically non-significant difference by unpaired two-tailed student’s *t*-test.

FIGURE S3 As exposure changes relative abundance of specific intestinal microbial taxa in HFD-fed mice. HFD-fed mice were exposed to 100 ppm As for 10 weeks (*n* = 6 for each group). Cecum fecal was collected for gut microbiota analysis by 16S rRNA gene sequencing. Phylogenetic tree created manually showing specific differences in intestinal microbial community at different taxonomic levels between As exposure and control mice. Nodes represent taxa, and the size of each node represents its relative abundance. The color red indicates an increase, blue indicates a decrease, and black indicates no change of relative abundance in As exposure compared with control mice. The full color of the nodes indicates the statistically significant difference and the hollow nodes indicate the statistically non-significant difference by unpaired two-tailed student’s *t*-test.

FIGURE S4 Cd exposure changes relative abundance of specific intestinal microbial taxa in ND-fed mice. ND-fed mice were exposed to 100 ppm Cd for 10 weeks (*n* = 6 for each group). Cecum fecal was collected for gut microbiota analysis by 16S rRNA gene sequencing. Phylogenetic tree created manually showing specific differences in intestinal microbial community at different taxonomic levels between Cd exposure and control mice. Nodes represent taxa, and the size of each node represents its relative abundance. The color red indicates an increase, blue indicates a decrease, and black indicates no change of relative abundance in Cd exposure compared with control mice. The full color of the nodes indicates the statistically significant difference and the hollow nodes indicate the statistically non-significant difference by unpaired two-tailed student’s *t*-test.

FIGURE S5 Cd exposure changes relative abundance of specific intestinal microbial taxa in HFD-fed mice. HFD-fed mice were exposed to 100 ppm Cd for 10 weeks (*n* = 6 for each group). Cecum fecal was collected for gut microbiota analysis by 16S rRNA gene sequencing. Phylogenetic tree created manually showing specific differences in intestinal microbial community at different taxonomic levels between Cd exposure and control mice. Nodes represent taxa, and the size of each node represents its relative abundance. The color red indicates an increase, blue indicates a decrease, and black indicates no change of relative abundance in Cd exposure compared with control mice. The full color of the nodes indicates the statistically significant difference and the hollow nodes indicate the statistically non-significant difference by unpaired two-tailed student’s *t*-test.

FIGURE S6 Pb exposure changes relative abundance of specific intestinal microbial taxa in ND-fed mice. ND-fed mice were exposed to 100 ppm Pb for 10 weeks (*n* = 6 for each group). Cecum fecal was collected for gut microbiota analysis by 16S rRNA gene sequencing. Phylogenetic tree created manually showing specific differences in intestinal microbial community at different taxonomic levels between Pb exposure and control mice. Nodes represent taxa, and the size of each node represents its relative abundance. The color red indicates an increase, blue indicates a decrease, and black indicates no change of relative abundance in Pb exposure compared with control mice. The full color of the nodes indicates the statistically significant difference and the hollow nodes indicate the statistically non-significant difference by unpaired two-tailed student’s *t*-test.

FIGURE S7 Pd exposure changes relative abundance of specific intestinal microbial taxa in HFD-fed mice. HFD-fed mice were exposed to 100 ppm Pb for 10 weeks (*n* = 6 for each group). Cecum fecal was collected for gut microbiota analysis by 16S rRNA gene sequencing. Phylogenetic tree created manually showing specific differences in intestinal microbial community at different taxonomic levels between Pb exposure and control mice. Nodes represent taxa, and the size of each node represents its relative abundance. The color red indicates an increase, blue indicates a decrease, and black indicates no change of relative abundance in Pb exposure compared with control mice. The full color of the nodes indicates the statistically significant difference and the hollow nodes indicate the statistically non-significant difference by unpaired two-tailed student’s *t*-test.

TABLE S1 Diet for Mice.

TABLE S2 Summary of microbiota changed by heavy metals.
