# Supplementary figures and images for: High-Fat Diet Affects Heavy Metal Accumulation and Toxicity to Mice Liver and Kidney Probably via Gut Microbiota
Source: Front Microbiol. 2020 Jul 28;11:1604. doi: 10.3389/fmicb.2020.01604 (PMC7399142; doi:10.3389/fmicb.2020.01604)

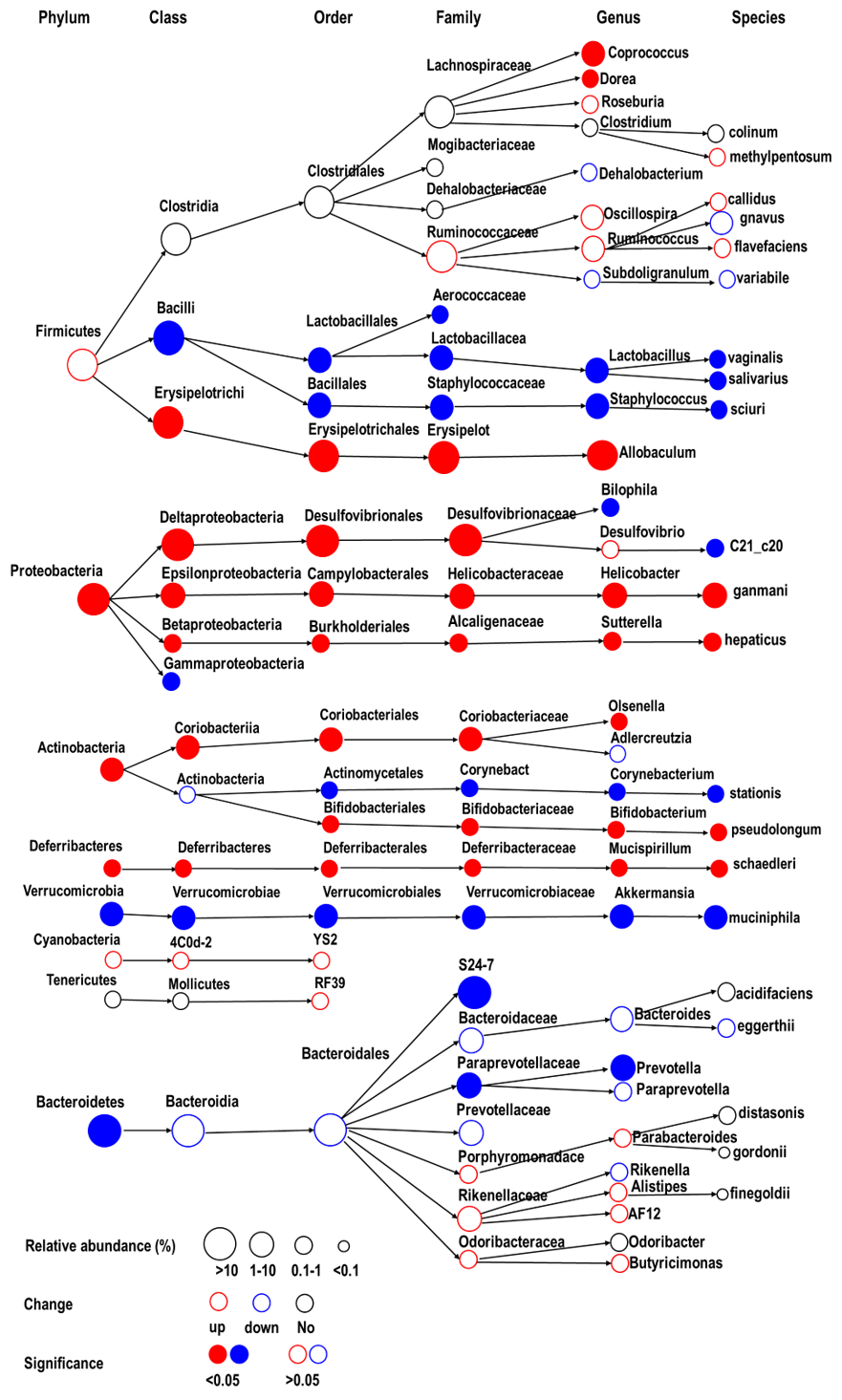

Supplement: FIGURE S1 — HFD changes relative abundance of most intestinal microbial taxa. Cecum fecal of ND- and HFD-fed mice (n = 6 for each group) was collected for gut microbiota analysis by 16S rRNA gene sequencing. Phylogenetic tree created manually showing specific differences in intestinal microbial community at different taxonomic levels between ND and HFD-fed mice. Nodes represent taxa, and the size of each node represents its relative abundance. The color red indicates an increase, blue indicates a decrease, and black indicates no change of relative abundance in HFD compared with ND fed mice. The fully colored nodes indicate the statistically significant difference (P < 0.05) and the hollow nodes indicate the statistically non-significant difference by unpaired two-tailed student’s t-test. [file Image_1.PNG]

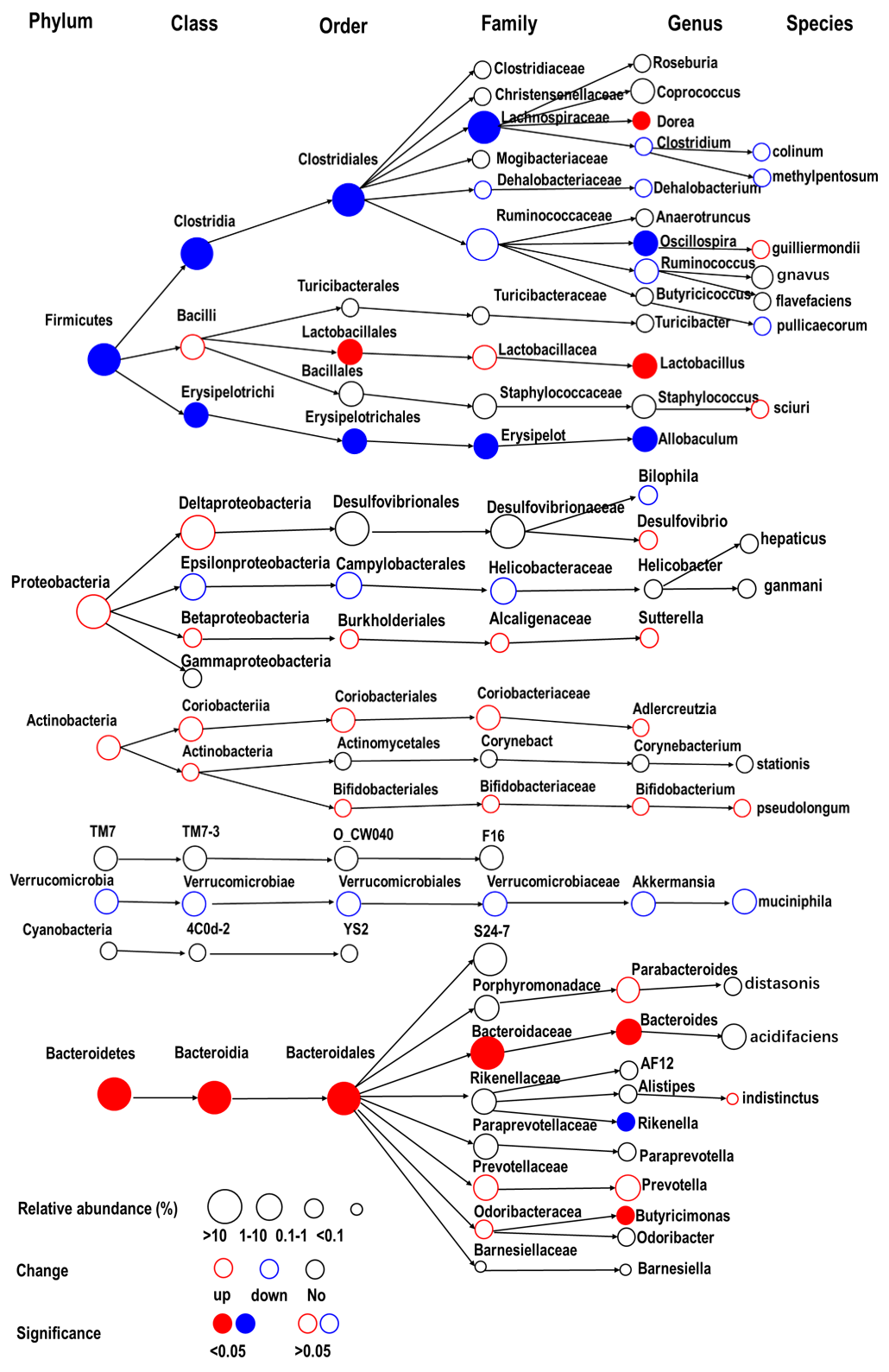

Supplement: FIGURE S2 — As exposure changes relative abundance of specific intestinal microbial taxa in ND-fed mice. ND-fed mice were exposed to 100 ppm As for 10 weeks (n = 6 for each group). Cecum fecal was collected for gut microbiota analysis by 16S rRNA gene sequencing. Phylogenetic tree created manually showing specific differences in intestinal microbial community at different taxonomic levels between As exposure and control mice. Nodes represent taxa, and the size of each node represents its relative abundance. The color red indicates an increase, blue indicates a decrease, and black indicates no change of relative abundance in As exposure compared with control mice. The full color of the nodes indicates the statistically significant difference and the hollow nodes indicate the statistically non-significant difference by unpaired two-tailed student’s t-test. [file Image_2.PNG]

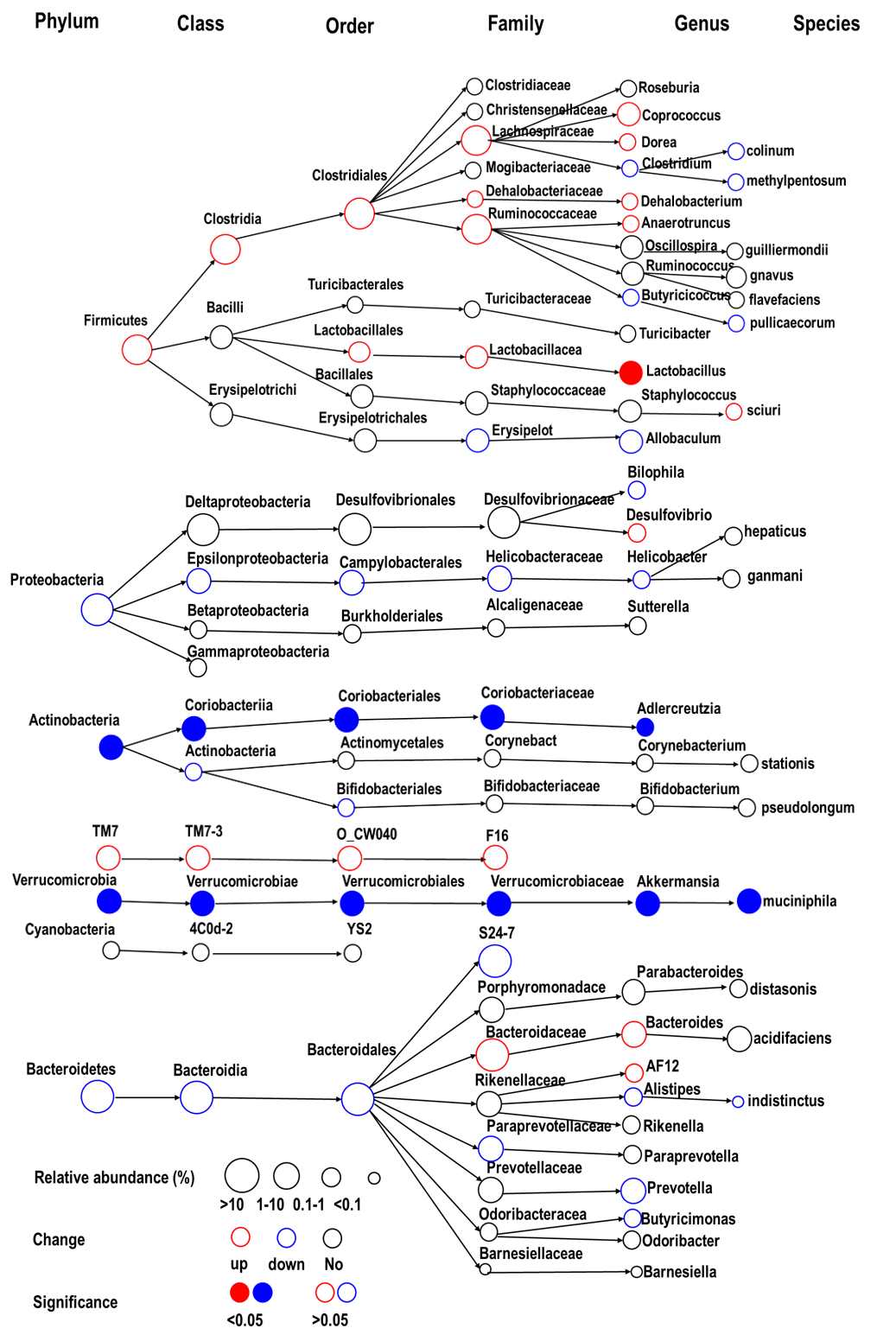

Supplement: FIGURE S3 — As exposure changes relative abundance of specific intestinal microbial taxa in HFD-fed mice. HFD-fed mice were exposed to 100 ppm As for 10 weeks (n = 6 for each group). Cecum fecal was collected for gut microbiota analysis by 16S rRNA gene sequencing. Phylogenetic tree created manually showing specific differences in intestinal microbial community at different taxonomic levels between As exposure and control mice. Nodes represent taxa, and the size of each node represents its relative abundance. The color red indicates an increase, blue indicates a decrease, and black indicates no change of relative abundance in As exposure compared with control mice. The full color of the nodes indicates the statistically significant difference and the hollow nodes indicate the statistically non-significant difference by unpaired two-tailed student’s t-test. [file Image_3.PNG]

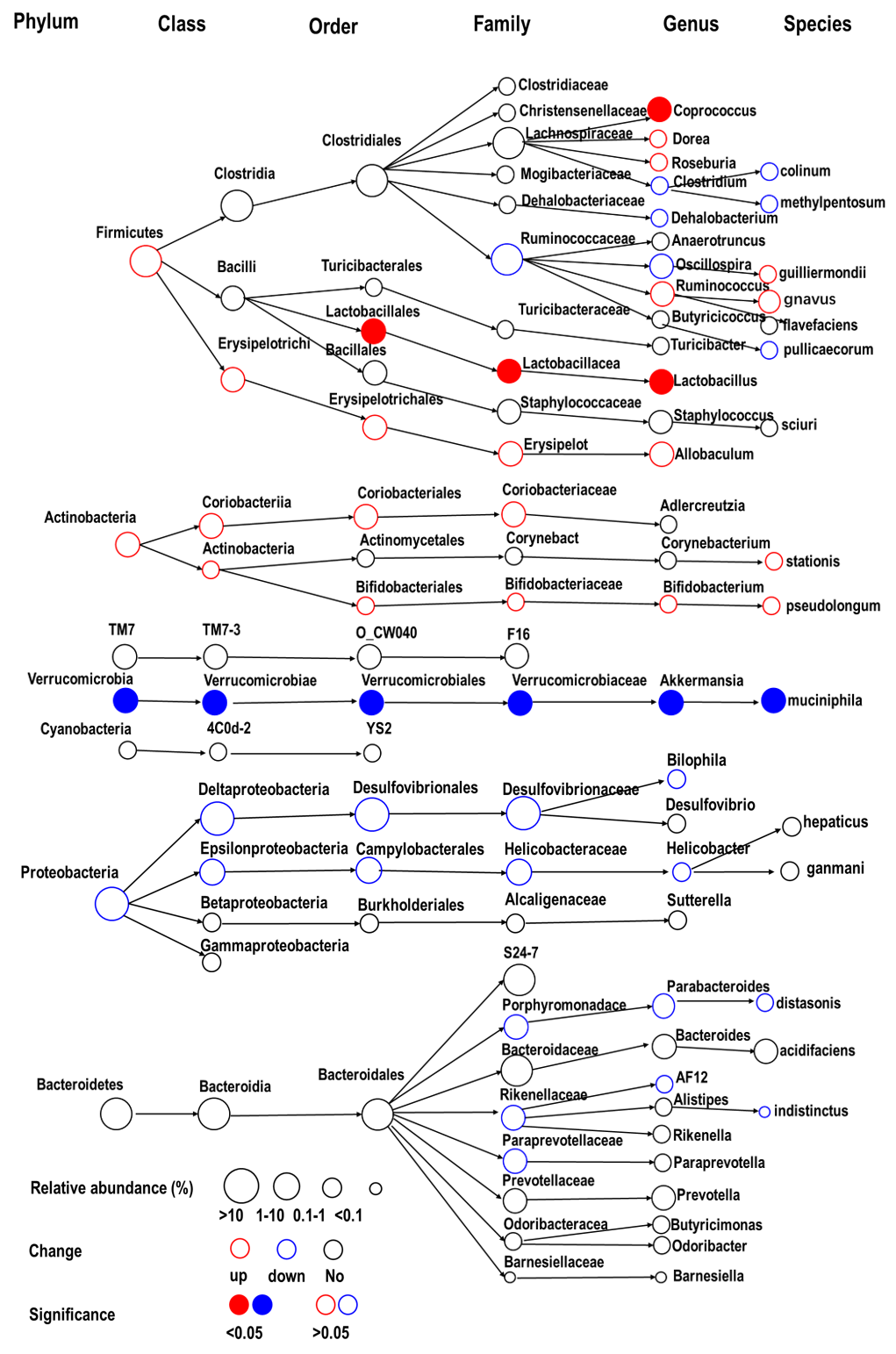

Supplement: FIGURE S4 — Cd exposure changes relative abundance of specific intestinal microbial taxa in ND-fed mice. ND-fed mice were exposed to 100 ppm Cd for 10 weeks (n = 6 for each group). Cecum fecal was collected for gut microbiota analysis by 16S rRNA gene sequencing. Phylogenetic tree created manually showing specific differences in intestinal microbial community at different taxonomic levels between Cd exposure and control mice. Nodes represent taxa, and the size of each node represents its relative abundance. The color red indicates an increase, blue indicates a decrease, and black indicates no change of relative abundance in Cd exposure compared with control mice. The full color of the nodes indicates the statistically significant difference and the hollow nodes indicate the statistically non-significant difference by unpaired two-tailed student’s t-test. [file Image_4.PNG]

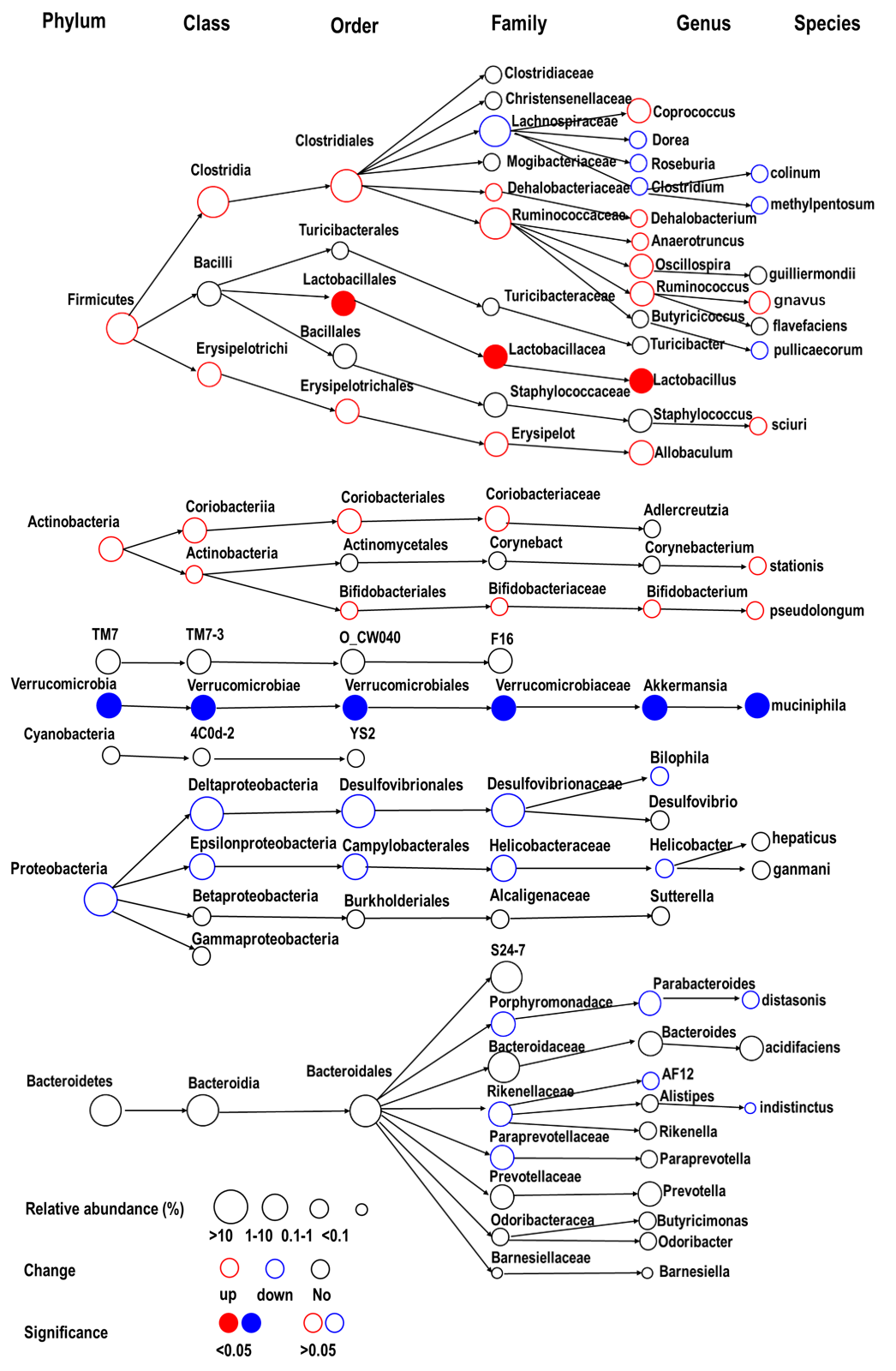

Supplement: FIGURE S5 — Cd exposure changes relative abundance of specific intestinal microbial taxa in HFD-fed mice. HFD-fed mice were exposed to 100 ppm Cd for 10 weeks (n = 6 for each group). Cecum fecal was collected for gut microbiota analysis by 16S rRNA gene sequencing. Phylogenetic tree created manually showing specific differences in intestinal microbial community at different taxonomic levels between Cd exposure and control mice. Nodes represent taxa, and the size of each node represents its relative abundance. The color red indicates an increase, blue indicates a decrease, and black indicates no change of relative abundance in Cd exposure compared with control mice. The full color of the nodes indicates the statistically significant difference and the hollow nodes indicate the statistically non-significant difference by unpaired two-tailed student’s t-test. [file Image_5.PNG]

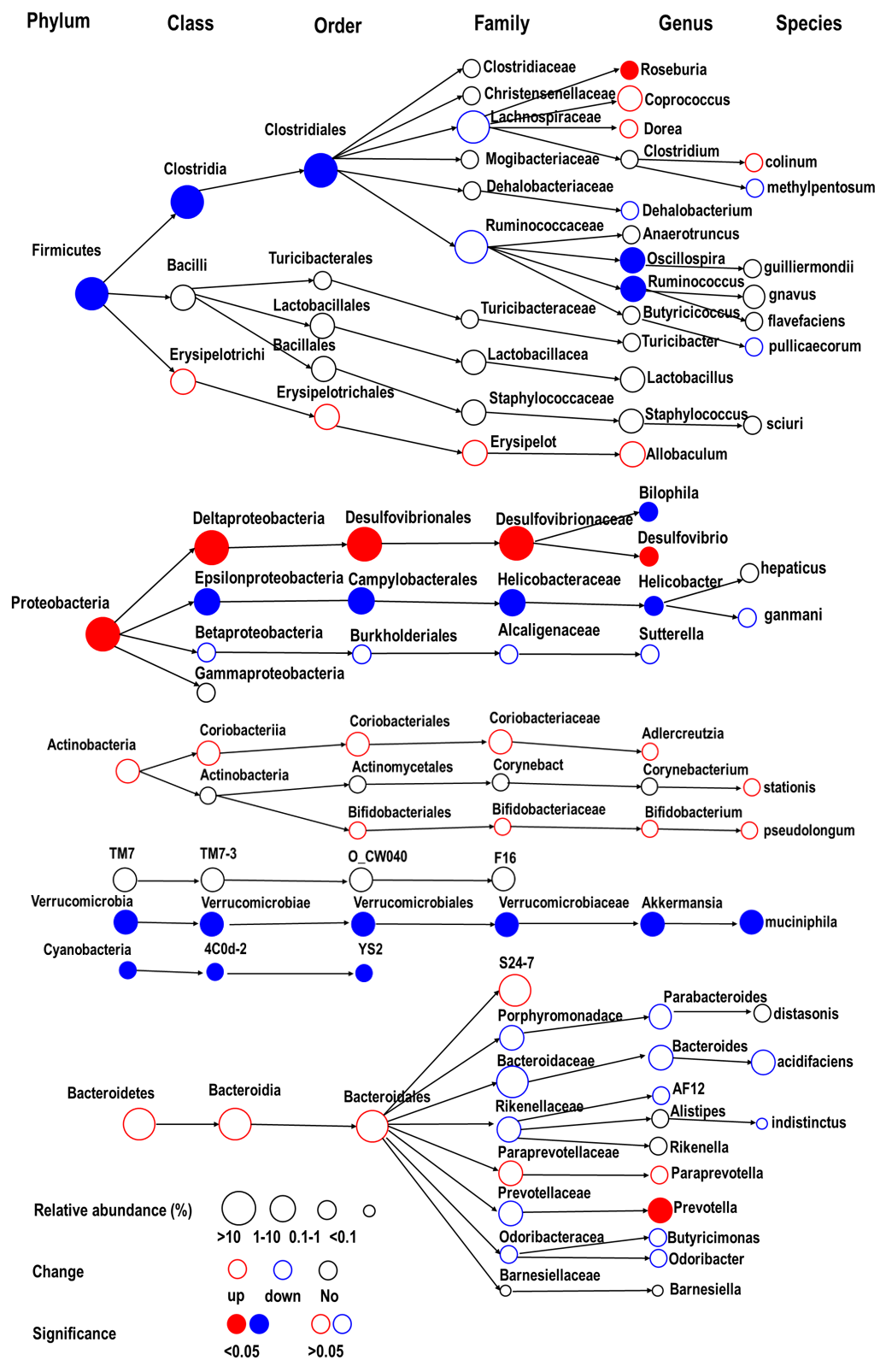

Supplement: FIGURE S6 — Pb exposure changes relative abundance of specific intestinal microbial taxa in ND-fed mice. ND-fed mice were exposed to 100 ppm Pb for 10 weeks (n = 6 for each group). Cecum fecal was collected for gut microbiota analysis by 16S rRNA gene sequencing. Phylogenetic tree created manually showing specific differences in intestinal microbial community at different taxonomic levels between Pb exposure and control mice. Nodes represent taxa, and the size of each node represents its relative abundance. The color red indicates an increase, blue indicates a decrease, and black indicates no change of relative abundance in Pb exposure compared with control mice. The full color of the nodes indicates the statistically significant difference and the hollow nodes indicate the statistically non-significant difference by unpaired two-tailed student’s t-test. [file Image_6.PNG]

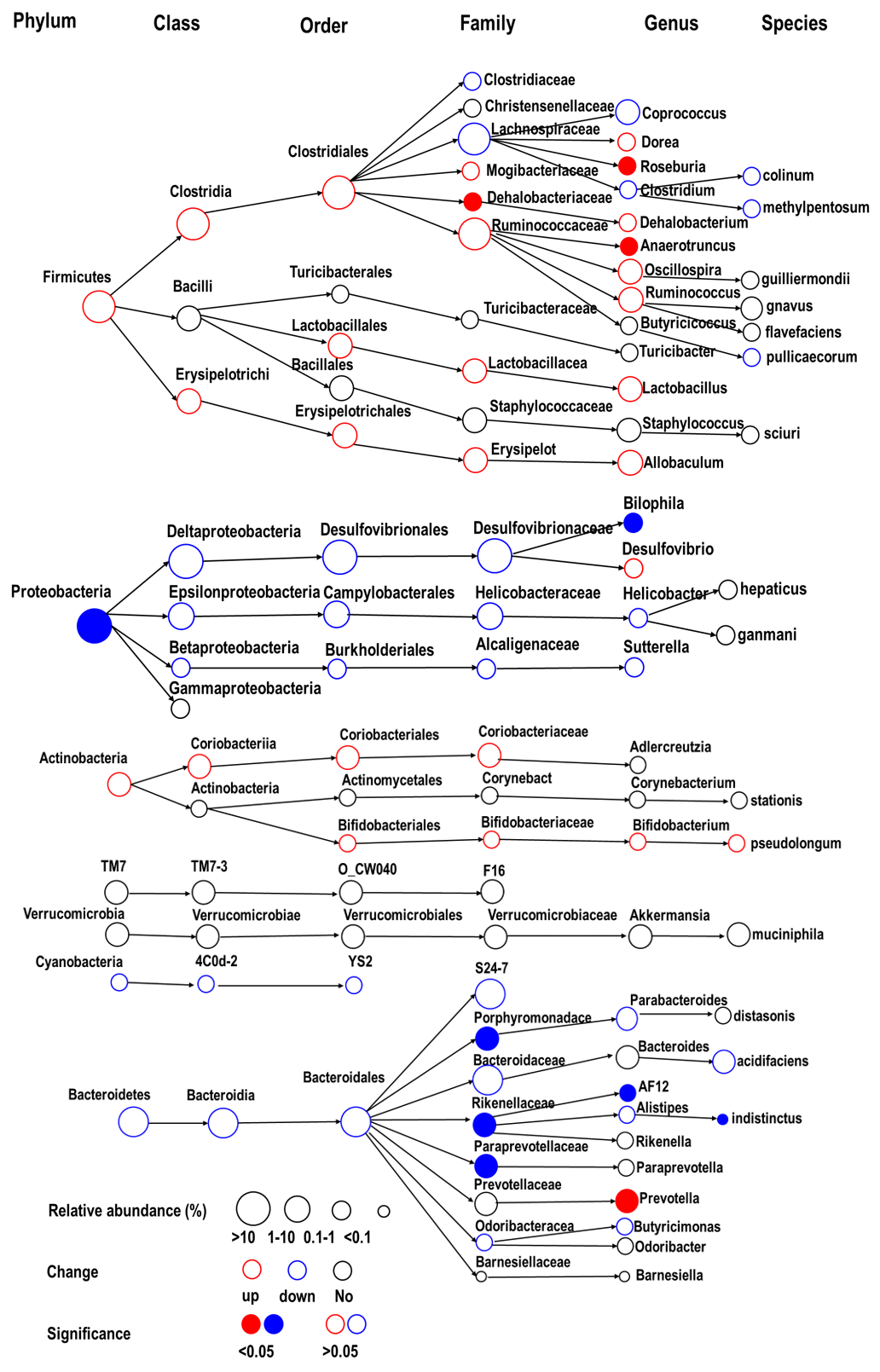

Supplement: FIGURE S7 — Pd exposure changes relative abundance of specific intestinal microbial taxa in HFD-fed mice. HFD-fed mice were exposed to 100 ppm Pb for 10 weeks (n = 6 for each group). Cecum fecal was collected for gut microbiota analysis by 16S rRNA gene sequencing. Phylogenetic tree created manually showing specific differences in intestinal microbial community at different taxonomic levels between Pb exposure and control mice. Nodes represent taxa, and the size of each node represents its relative abundance. The color red indicates an increase, blue indicates a decrease, and black indicates no change of relative abundance in Pb exposure compared with control mice. The full color of the nodes indicates the statistically significant difference and the hollow nodes indicate the statistically non-significant difference by unpaired two-tailed student’s t-test. [file Image_7.PNG]
