# Supplementary material for: High-Fat Diet Affects Heavy Metal Accumulation and Toxicity to Mice Liver and Kidney Probably via Gut Microbiota
Source: Front Microbiol. 2020 Jul 28;11:1604. doi: 10.3389/fmicb.2020.01604 (PMC7399142; doi:10.3389/fmicb.2020.01604)
Supplement: TABLE S1 — Diet for Mice. [file Table_1.DOCX]

**Table S1 Diet for Mice**

|  |  | Normal Diet | High fat diet |
| --- | --- | --- | --- |
| Ingredients (g/kg) | Casein | 200 | 200 |
|  | L-cysteine | 3 | 3 |
|  | Corn starch | 315 | 72.8 |
|  | Maltodextrin | 35 | 100 |
|  | sucrose | 350 | 172.8 |
|  | Crude fiber | 50 | 50 |
|  | soybean oil | 25 | 25 |
|  | lard oil | 20 | 177.5 |
|  | Mineral mix | 35 | 35 |
|  | Vitamin mix | 10 | 10 |
|  | choline chloride | 2.5 | 2.5 |
| Composition (kcal%) | Protein | 20 | 20 |
|  | Carbohydrates | 70 | 35 |
|  | Fat | 10 | 45 |

**Table S2 Summary of microbiota changed by heavy metals**

| Microbiota  (Genus level) | Abundance Change | | | | | |
| --- | --- | --- | --- | --- | --- | --- |
|  | As exposure | | Cd exposure | | Pb exposure | |
|  | ND | HFD | ND | HFD | ND | HFD |
| *Dorea* | **↑** | ↑ | ↑ | ↓ | ↑ | ↑ |
| *Lactobacillus* | **↑** | **↑** | **↑** | **↑** | ― | ↑ |
| *Bacteroides* | **↑** | ↑ | ― | ― | ↓ | ― |
| *Butyricimonas* | **↑** | ↓ | ― | ― | ― | ― |
| *Allobaculum* | **↓** | ↓ | ↑ | ↑ | ↑ | ↑ |
| *Oscillospira* | **↓** | ― | ↓ | ↑ | ↓ | ↑ |
| *Rikenella* | **↓** | ― | ― | ― | ― | ― |
| *Adlercreutzia* | ↑ | **↓** | ― | ― | ↑ | ― |
| *Akkermansia* | ↓ | **↓** | **↓** | **↓** | **↓** | ― |
| *Coprococcus* | ― | ↑ | **↑** | ↑ | ↑ | ↓ |
| *Roseburia* | ― | ― | ↑ | ↓ | **↑** | **↑** |
| *Desulfovibrio* | ↑ | ↑ | ― | ― | **↑** | ↑ |
| *Prevotella* | ↑ | ↓ | ― | ― | **↑** | **↑** |
| *Ruminococcus* | ↓ | ― | ↑ | ↑ | **↓** | ↑ |
| *Bilophila* | ↓ | ↓ | ↓ | ↓ | **↓** | **↓** |
| *AF12* | ― | ↑ | ↓ | ↓ | ↓ | **↓** |

The red arrows indicated an increase, blue represented a decrease and black horizontal line means no change of relative abundance in heavy metal challenged microbiota compared with their controls. The bold color of the arrows indicated the statistically significant difference (P<0.05) and the hollow ones indicated the statistically non-significant difference by unpaired two-tailed student’s t-test.
